# Supplementary material for: Improving the safety of outpatient to Emergency Department transfers: A quality improvement study in a tertiary hospital in Pakistan
Source: PLoS One. 2025 Nov 5;20(11):e0336347. doi: 10.1371/journal.pone.0336347 (PMC12588536; doi:10.1371/journal.pone.0336347)
Supplement: S1 File — (DOCX) [file pone.0336347.s001.docx]

**Transfer Form**

**From Ambulatory Services**

**Date: _________ Time: _____________**

**Transferred from: (Please tick one box and mention specialty)**

NZW ____________________ CC _______________________

IBZ _____________________

**Area to be transferred to:**

Emergency Department Urgent Care Services Others____________

**Mode of transportation:**

Wheelchair Stretcher Ambulatory Others___________

**Patient’s Hands-off at ED/ unit**

**Situation:**

Presenting Complain: Diagnosis: _____________________

Surgical Procedure (if any):______________________________________________________

**Background:**

Comorbid/known case (if any) ___________________________

Others __________________

Infection status: Contact Precaution Droplet Precaution Airborne Precaution Not Known

**Assessment:**

Alert Response to voice Response to pain Unresponsive

**Vital Signs:**

B.P ________mmHg H/R: _____/min R/R: _____/min SPO2: ______% Temp: _______ C

Spontaneous breathing Supplemental Oxygen

Pain Score: ____________ Intravenous Line: _______________ Tubes/Drains: _____________

**Recommendation:**

Plan of Care: __________________________________________________________________

Any other comments: ____________________________________________________________

Handover given by: ____________________________________________________________

Handover received by: __________________________________________________________
