# Supplementary material for: Improving the safety of outpatient to Emergency Department transfers: A quality improvement study in a tertiary hospital in Pakistan
Source: PLoS One. 2025 Nov 5;20(11):e0336347. doi: 10.1371/journal.pone.0336347 (PMC12588536; doi:10.1371/journal.pone.0336347)
Supplement: Attachment — Submitted filename: Response to Reviewers.docx [file pone.0336347.s006.docx]

**Response to Reviewer**

**Subject: Revised Manuscript Submission – PONE-D-25-12396**

Dear Dr. Mohd Ismail Ibrahim,

Thank you for considering our manuscript (ID: PONE-D-25-12396), entitled “**Rapid response and safety optimization: enhancing critical transfers from outpatient clinics to high acuity areas – a quality improvement project**” for review.

We are grateful for your and the reviewers’ insightful comments. In response, we have carefully revised and modified our manuscript in accordance with the reviewers' suggestions. We believe these revisions have strengthened the manuscript and hope that it is now suitable for publication in PLOS ONE.

In accordance with the journal's guidelines, we are submitting the following files:

- A marked-up copy of the revised manuscript with tracked changes, labeled **"Revised Manuscript with Track Changes"**.
- A clean, unmarked version of the revised manuscript, labeled **"Manuscript"**.

We appreciate your time and consideration, and we remain available to address any further questions or concerns you may have.

Yours sincerely,
On behalf of all authors,
**Dr. Samar Fatima**

| **Reviewer’s comments** | **Authors’ response** |
| --- | --- |
| **ACADEMIC EDITOR** | |
| Add a short paragraph in the Methods section noting the lack of control group, which is only mentioned later in the Limitations. | As per your suggestion, the following paragraph has been added to the **Methods** section under the heading Study Design, Setting, and Data Source, addressing the absence of a control group.  For ease of reference, the paragraph is provided below:  " A control group was not included in the study due to the operational and ethical challenges of withholding a potentially beneficial intervention from a subset of patients and staff. Given the quality and safety implications associated with patient transfers, it was deemed necessary to implement the intervention across the entire relevant cohort to ensure standardized practice and minimize risk." |
| Clarify whether the handoff tool was validated (face/content validity, pilot feedback, etc.) before implementation | The handoff tool was validated prior to implementation using a modified Delphi method to establish content validity. It was piloted during Phase One of the study. Feedback from this pilot led to minor revisions to enhance clarity and usability before final implementation. The Phase One section in the manuscript has also been revised for improved clarity. |
| While p-values are provided, effect sizes for pre-post test differences should be added to convey practical significance. | We appreciate the reviewer's insightful comment. In response, we have incorporated Cohen's d values in Table 5 to complement the p-values. Effect sizes provide an important metric for understanding the magnitude of differences beyond statistical significance, reinforcing the practical implications of our findings.  We believe this addition enhances the interpretability of our results and supports a more comprehensive discussion of the observed changes. |
| Explicitly state how missing data (e.g., 2.28% in Table 7) was handled in analysis. | We appreciate the reviewer's request for clarification regarding the handling of the missing data. To assess the randomness of missing values, we conducted Little’s MCAR test, which evaluates whether data are missing completely at random (MCAR) details are added under the heading of “Statistical Analysis”. The test results (χ² = 4.8987, p = 0.2979) confirmed that the missingness was random and did not introduce bias in our analysis. Given this confirmation, the missing data were handled appropriately without requiring further imputation or adjustments, ensuring the integrity of our statistical conclusions (also added in the statistical analysis section of the manuscript) |
| Figures (1–4) are referenced but were not included in the PDF for evaluation (they are in separate TIFF files). Ensure that these are reviewed and captions are descriptive enough to be interpreted independently. | Thank you for your observation. When uploading, we will ensure that all figures are included within the PDF file for evaluation.  We have also reviewed the figures and provided descriptive captions to ensure they can be interpreted independently.  To clarify, two figures have been added for Phase One and one figure for Phase Two (as per the comment of Reviewer # 1). Additionally, the previously labeled Figures 1 and 2 have been replaced with tables to improve clarity and presentation.  As per PLOS ONE guidelines, figures are uploaded separately and are automatically included at the end of the compiled PDF file. We have also reviewed our previous submission, where the figures are appearing in the PDF after the main manuscript and before the supplementary material. |
| The handoff tool (S1 File) should be briefly described (e.g., sections, items) in the text when first introduced. | We have now included a brief description of the handoff tool (S1 File) in the text at its first mention. |
| Minor polishing is needed for consistent tenses and clarity. For example: "Patients were predominantly transported by stretcher (48.13%) or walking (34.7%)" → "on foot" might be clearer. “...hospital-wide over the next two to three months” → specify actual timeline if possible. | We have reviewed the manuscript to ensure consistent use of tenses and improved clarity throughout. |
| Strengthen discussion with comparative insights from other QI projects in LMICs or outpatient-to-ED transition studies, if available. | We found limited published data specifically addressing quality improvement initiatives focused on the transition of patients from outpatient clinics to emergency departments or other high acuity areas, particularly in low- and middle-income countries (LMICs).  To address this gap, we identified and included three pertinent studies that describe different models implemented within outpatient settings aimed at improving the management and transfer of critically ill patients to higher acuity care areas. These studies provide valuable frameworks for understanding effective strategies, challenges, and outcomes related to such transitions.  In the Discussion section, we have integrated these studies to provide comparative insights, highlighting similarities and differences with our intervention. |
| Clarify whether the training program has ongoing refresher modules or sustainability plans post-study. | We have added a new section titled “Sustainability Measures and Ongoing Staff Competency Reinforcement” after the phases.  The training program is now part of the orientation for new nurses, and quarterly refresher sessions are held for existing staff to maintain key skills related to early warning signs, the handoff tool, and patient transfer protocols. |
| If you would like to make changes to your financial disclosure, please include your updated statement in your cover letter | We have reviewed our financial disclosures and confirm that there are no changes. We have nothing to disclose and stated this in the cover letter in bold. |
| If applicable, we recommend that you deposit your laboratory protocols in protocols.io to enhance the reproducibility of your results. | Our study did not involve laboratory protocols requiring deposition in protocols.io. |
| **Journal Requirement** | |
| Please ensure that your manuscript meets PLOS ONE's style requirements, including those for file naming. | We have reviewed and updated the manuscript and all associated files to ensure they meet PLOS ONE’s style and file naming requirements. |
| We note that your Data Availability Statement is currently as follows: [All relevant data are within the manuscript and its Supporting Information files.]. Please confirm at this time whether your submission contains all raw data required to replicate the results of your study. Authors must share the “minimal data set” for their submission. PLOS defines the minimal data set to consist of the data required to replicate all study findings reported in the article, as well as related metadata and methods (https://journals.plos.org/plosone/s/data-availability#loc-minimal-data-set-definition). | We are uploading the required data set with this revision under the file titled **“Data Set”**, in accordance with PLOS ONE guidelines.  There will be two excel files: one for pre- and post-test data, and another for data on transferred patient outcomes |
| Please review your reference list to ensure that it is complete and correct. If you have cited papers that have been retracted, please include the rationale for doing so in the manuscript text or remove these references and replace them with relevant current references. Any changes to the reference list should be mentioned in the rebuttal letter that accompanies your revised manuscript. | We have reviewed the reference list and confirm that it is complete and accurate. None of the cited papers have been retracted.  However, References 1 and 9, which were previously sourced from internet-based materials, have been replaced with citations from peer-reviewed journal articles for greater reliability. |
| While revising your submission, please upload your figure files to the Preflight Analysis and Conversion Engine (PACE) digital diagnostic tool, [https://pacev2.apexcovantage.com/](https://eur03.safelinks.protection.outlook.com/?url=https%3A%2F%2Ftrack.editorialmanager.com%2FCL0%2Fhttps%3A%252F%252Fpacev2.apexcovantage.com%252F%2F1%2F010f0196887f602e-25d15613-e756-49a8-abeb-3e346822b840-000000%2FJbdhvt4AqezdixZ2Mfm4688fy2QqFt3b09fdwG2kjd8%3D208&data=05%7C02%7Csamar.fatima%40aku.edu%7Cc89e6339a6114fc6af5a08dd8829e828%7Ca5d4252a02f94e6096f09733baae4919%7C0%7C0%7C638816436076720077%7CUnknown%7CTWFpbGZsb3d8eyJFbXB0eU1hcGkiOnRydWUsIlYiOiIwLjAuMDAwMCIsIlAiOiJXaW4zMiIsIkFOIjoiTWFpbCIsIldUIjoyfQ%3D%3D%7C0%7C%7C%7C&sdata=18Duh2ONqy4C8N9F6FHIbrLqsFmMDtxtV1lFovDhbbI%3D&reserved=0). PACE helps ensure that figures meet PLOS requirements. To use PACE, you must first register as a user. Registration is free. Then, login and navigate to the UPLOAD tab, where you will find detailed instructions on how to use the tool. If you encounter any issues or have any questions when using PACE, please email PLOS at [figures@plos.org](mailto:figures@plos.org). Please note that Supporting Information files do not need this step. | Thank you for the guidance. We have uploaded the figure files to the PACE tool as instructed and have ensured they comply with PLOS formatting requirements. |
| **Reviewer 1** | |
| Title Improvement Needed: The title of the manuscript could be enhanced for clarity and relevance. | We sincerely appreciate your valuable suggestion regarding the clarity and relevance of the manuscript title. In response, we have revised the title to better reflect the study’s objectives and scope. The original title,  **"Rapid response and safety optimization: enhancing critical transfers from outpatient clinics to high acuity areas – a quality improvement project,"**  has been replaced with:  **"Ensuring Safe Transfer of Critically Ill Patients from Outpatient Clinics to High-Acuity Areas: A Quality Improvement Project."**  We hope this revised title aligns better with the journal’s standards and expectations. |
| Abstract Method Clarity: The methodology is not clearly articulated in the abstract. A more precise definition of the phases and techniques used is necessary. | In response to the request for greater clarity in the abstract methodology section, we have revised it to clearly define the study’s two phases, including the methods employed, timelines, and evaluation processes. This revision aims to adequately address the concern and improve overall clarity. |
| Introduction Problem Justification: The significance of the research problem should be elaborated upon. A clearer explanation of why this study is necessary would strengthen the introduction. | We have expanded the introduction to provide a more detailed justification of the research problem, emphasizing the significance and necessity of this study. This enhancement aims to strengthen rationale and better contextualize the importance of our work. |
| Methodology General Explanation: A general overview of the phases should be presented in a dedicated figure within this section. | We have added dedicated figures in the Methodology section that provides a clear overview of the study phases to enhance reader comprehension. |
| Method Explanation: The overall methodology lacks clarity. A more thorough explanation is needed to understand the study's design and execution. | We have revised the Methodology section to provide a more detailed and clear explanation of the study design and execution. |
| Discussion Findings-Based Discussion: The discussion section should initiate with a summary of the primary findings from the study.  Comparative Analysis: Include comparisons with countries having similar income levels or employing analogous methods, such as India and Iran. This context would enrich the discussion and provide a broader perspective. | We began the Discussion section with a concise summary of the primary findings of our study. To the best of our knowledge and following a thorough literature search, we did not find any studies from low- and middle-income countries (LMICs) or neighboring countries that specifically address quality improvement initiatives focused on patient transitions from outpatient clinics to emergency or high-acuity areas relevant to our study title.  To address this gap, we identified and incorporated three relevant studies describing models used in outpatient settings to improve the management and transfer of critically ill patients to higher acuity care. These studies provide useful frameworks for understanding effective strategies, challenges, and outcomes in similar contexts.  These comparative insights have been integrated into the Discussion section to provide broader context and enrich the interpretation of our findings. |
| Presentation Schematic Representation: Processes should be illustrated in a schematic form to aid understanding. | We have revised the manuscript according to your recommendation by adding a dedicated figure in the Methods section that provides a clear overview of the study phases and workflow. |
| Conclusion Ethical Declaration: A concluding declaration should be added to address ethics, budget considerations, author contributions, acknowledgments, and any necessary thanks. | In response to your comment, the following have been addressed in the revised manuscript:   - A patient and public involvement declaration has been added after the Methods section as ethical declaration. - Author contributions have been detailed separately in accordance with journal guidelines. - An Acknowledgments section has been included in the revised manuscript. |
