# Supplementary material for: Improving the safety of outpatient to Emergency Department transfers: A quality improvement study in a tertiary hospital in Pakistan
Source: PLoS One. 2025 Nov 5;20(11):e0336347. doi: 10.1371/journal.pone.0336347 (PMC12588536; doi:10.1371/journal.pone.0336347)
Supplement: Attachment — Submitted filename: S Nontamo_PLOS ONE Review.docx [file pone.0336347.s005.docx]

**General Comments**

This is a well-organized and useful Quality Improvement (QI) project that aimed to improve the safety and effectiveness of patient flow from outpatient clinics to the ED at a tertiary care setting in Pakistan. The subject is especially pertinent in low- and middle-income countries (LMICs), where healthcare systems operate under varying challenges specific to each setting. The authors discuss a topic that is often overlooked when considering patient safety, and the interventions described are relatively pragmatic and potentially scalable. However, there are several areas where improvements would enhance the manuscript's scientific rigor, clarity, and impact.

**Specific Comments**

1. **Title & Abstract**

- The title is well structured, but could be concise, such as

*“Improving the Safety of Outpatient to Emergency Department Transfers: A Quality Improvement Study in a Tertiary Hospital in Pakistan”*

- The abstract follows a logical structure, yet needs keywords like **quality improvement,** **handover,** and **patient safety** to improve search results

2. **Introduction**

- The similar initiatives as part of the literature supporting the relevance of your study were not cited sufficiently, such as QI initiatives in similar LMIC settings (abroad and local).
- Please look for studies in QI Initiatives conducted in similar settings to support your argument.

- Reword or rephrase for conciseness and impact, maybe to write:

*‘Transfers from outpatient clinics to the Emergency Department are vulnerable points in the care continuum, especially in low and middle-income countries, where the standardized protocols are often lacking’*

- In this introduction, the problem and its significance were not clearly defined.
- Please define clearly the problem and its significance.
- Secondly, support your problem by mentioning the available data on occurrences, like noting that:

*‘? % of patients reported missing paperwork…’*

- It is always recommended that your introduction ends with the purpose or aim of conducting the study, e.g.:
  *"This study aimed to improve the safety, documentation, and tracking of patients transferred from outpatient clinics to the ED through a quality improvement approach at Aga Khan University Hospital.*

3. **Methods**

**3.1 Study Design and Setting**

This is not a clinical trial. Therefore, mention that this is a QI initiative to minimize confusion for the readers.

When conducting a Quality Improvement study in healthcare, the SQUIRE 2.0 (Standards for Quality Improvement Reporting Excellence) is often used for reporting.

SQUIRE 2.0 provides a framework for authors to report their QI projects clearly and transparently.

Therefore, I recommend that you use the SQUIRE 2.0 structure to address the following 3 areas, namely;

a) ***Context***

Describe the hospital setting (for instance, yearly patient numbers, number of specialties)

b) ***Intervention rationale:***

Explain why these specific tools, such as the **transfer form** and **clinic attendant**, were chosen.

c) ***Measures:***

Lastly, using the SQUIRE 2.0 structure, define: ***process*** vs ***outcome*** vs ***balancing measures***.

**3.2 Intervention Design**

Though you expressed in detail the interventions applied in your study, it is unclear how you designed the interventions. Explain how you designed the interventions, such as Process mapping, Stakeholder meetings, and the PDSA cycle (PLAN-DO-STUDY-ACT)

**3.3 Data Collection and Measures**

- This research (QI Intervention study), referencing primary, secondary, and process measures, is required to understand the intervention's effectiveness.

In this case, primary measures (number of adverse events or patient harm during transfers), secondary measures (triage accuracy, length of ED stay, or patient satisfaction), and process measures (form usage, adherence to protocol, or documentation completeness)

Please address this shortcoming in your data collection section.

- The explanation of how the audits were conducted was also not provided.

It will be important to mention this in the data collection section.

4. **Ethics Statement**

Even though this is a QI study, PLOS ONE requires ethics info. You can write:

“*This quality improvement study was approved as a non-research activity by the Ethical Review Committee of Aga Khan University Hospital, which waived the need for informed consent as no patient-identifiable data was used.”*

It will also be important to mention the study code and the date on which the ethical clearance was issued.

In case you did not receive the ethical clearance, you must explain why you did not obtain formal ethics approval and under what guidelines it was exempted.

5. **Results**

Though the data was presented in table form extensively, to strengthen the QI focus, I recommend that you also include ***run charts*** or ***control charts***.

6. **Discussion**

In your first paragraph, summarize the main results of the study, followed by how your intervention brought about change.

- Though the discussion section articulated deeply on challenges, critical challenges such as staff resistance, staff turnover, buy-in, and form fatigue, among others, did not come out.

I therefore recommend that you add one paragraph to discuss these challenges explicitly.

- Since this is a QI intervention study, this study lacks comparison with other similar local or international published QI Intervention studies.

Therefore, I recommend that you compare your results with other published QI interventions in similar settings, such as the WHO Safer Handover toolkits or other LMIC QI initiatives. This comment is also mentioned in your introduction!

- It would be prudent to suggest explicitly the areas for further improvement, such as digital tools, automated alerts, and sustainability audits. This would ensure the continuity and improve future interventions.

Outline clearly the practical and actionable future activities that would enhance the safety of the outpatient during emergency transfers.

7. **Conclusion**

The current version is good, but consider making sustainability and scalability more explicit. Keep it concise and focused on impact by including the following phrase:

*“Structured communication, physical accompaniment, and stakeholder collaboration significantly improved the safety of patient transfers from outpatient clinics to the ED. This approach is low-cost, sustainable, and scalable to similar settings.”*
