# Supplementary material for: Improving the safety of outpatient to Emergency Department transfers: A quality improvement study in a tertiary hospital in Pakistan
Source: PLoS One. 2025 Nov 5;20(11):e0336347. doi: 10.1371/journal.pone.0336347 (PMC12588536; doi:10.1371/journal.pone.0336347)
Supplement: Attachment — Submitted filename: Response_to_Reviewers_auresp_2.docx [file pone.0336347.s008.docx]

**Response to Reviewer**

**Subject: Revised Submission – Manuscript ID PONE-D-25-12396R2**

Dear Dr. Mohd Ismail Ibrahim,

Thank you for your detailed feedback and for giving us more time to revise our manuscript. We are grateful to you and the reviewers for your constructive comments, which have helped us improve the clarity, rigor, and presentation of our work.

In response to the reviewers’ suggestions, we have:

- Addressed each comment in detail in the attached **“Response to Reviewers”** document.
- Revised the manuscript to incorporate all necessary changes, including methodological clarifications, improved discussion points, and updated references where appropriate.
- Provided both a **marked-up version** showing tracked changes and a **clean version** of the revised manuscript.

We believe the revisions have strengthened the manuscript and addressed all concerns raised during the review process. We appreciate your consideration of our revised submission and look forward to your feedback.

Kind regards,
Dr. Samar Fatima

**Journal Requirements**

**Comment 1:**

If you would like to make changes to your financial disclosure, please include your updated statement in your cover letter. Guidelines for resubmitting your figure files are available below the reviewer comments at the end of this letter.

**Reply 1:**

We would like to confirm that there have been no changes to the financial statements previously provided, as referenced in the cover letter.

All figure files have been checked to ensure they meet PLOS ONE submission requirements.

**Comment 2:**

If applicable, we recommend that you deposit your laboratory protocols in protocols.io to enhance the reproducibility of your results. Protocols.io assigns your protocol its own identifier (DOI) so that it can be cited independently in the future. For instructions, see: [https://journals.plos.org/plosone/s/submission-guidelines#loc-laboratory-protocols](https://eur03.safelinks.protection.outlook.com/?url=https%3A%2F%2Ftrack.editorialmanager.com%2FCL0%2Fhttps%3A%252F%252Fjournals.plos.org%252Fplosone%252Fs%252Fsubmission-guidelines%2523loc-laboratory-protocols%2F1%2F010f01987403cc37-990ac586-0170-4797-b5ef-0f380e1e1938-000000%2FKAjUWM5J-lPcUBAvvXKUD2zaOrS1Bm2k47FoGBZYyRQ%3D222&data=05%7C02%7Csamar.fatima%40aku.edu%7Cb94ec45fde4442616ea108ddd329ccde%7Ca5d4252a02f94e6096f09733baae4919%7C0%7C0%7C638898898816798998%7CUnknown%7CTWFpbGZsb3d8eyJFbXB0eU1hcGkiOnRydWUsIlYiOiIwLjAuMDAwMCIsIlAiOiJXaW4zMiIsIkFOIjoiTWFpbCIsIldUIjoyfQ%3D%3D%7C0%7C%7C%7C&sdata=xM9hGAvGVtTzy0mGUAOjCbgII0n5Uv7A4ln0lI9eKoo%3D&reserved=0). Additionally, PLOS ONE offers an option for publishing peer-reviewed Lab Protocol articles, which describe protocols hosted on protocols.io. Read more information on sharing protocols at [https://plos.org/protocols?utm_medium=editorial-email&utm_source=authorletters&utm_campaign=protocols](https://eur03.safelinks.protection.outlook.com/?url=https%3A%2F%2Ftrack.editorialmanager.com%2FCL0%2Fhttps%3A%252F%252Fplos.org%252Fprotocols%253Futm_medium%3Deditorial-email%2526utm_source%3Dauthorletters%2526utm_campaign%3Dprotocols%2F1%2F010f01987403cc37-990ac586-0170-4797-b5ef-0f380e1e1938-000000%2FIkTGWsai0iRpfS2aEIrNgZaNUHdYZUyPdWvWBasZmmA%3D222&data=05%7C02%7Csamar.fatima%40aku.edu%7Cb94ec45fde4442616ea108ddd329ccde%7Ca5d4252a02f94e6096f09733baae4919%7C0%7C0%7C638898898816833529%7CUnknown%7CTWFpbGZsb3d8eyJFbXB0eU1hcGkiOnRydWUsIlYiOiIwLjAuMDAwMCIsIlAiOiJXaW4zMiIsIkFOIjoiTWFpbCIsIldUIjoyfQ%3D%3D%7C0%7C%7C%7C&sdata=gZyGJBb88Te4B7503lWN5IfwRmXRPDHKxA9SwRAG3Rc%3D&reserved=0).

**Reply 2:**

Our study does not involve laboratory protocols requiring deposition in protocols.io. Therefore, this recommendation does not apply to the present manuscript.

**Reviewer #1**

**Comment:**

Dear authors, unfortunately, no changes have been made to the article, and the text has not been colored. This study has problems in the title, abstract, introduction, method, type of instrument, patient population, method of patient selection, and also in the selection of experts.

**Reply:**

In view of Reviewer 1’s above comment, we believe that there may have been a misunderstanding and that the reviewer may have inadvertently reviewed an older version of our manuscript rather than the revised submission with tracked changes.

We appreciate the Academic Editor’s review of our concerns and confirmation that substantial changes were made in response to Reviewer 1’s original feedback. We also acknowledge the Editor’s assessment that the comment “no changes have been made” does not align with the actual revisions incorporated, and that this may have resulted from difficulty accessing the track-changed document.

The link to the Academic Editor’s full email response confirming this matter has also been provided here for reference: [[Academic Editor Response](https://aku4-my.sharepoint.com/personal/samar_fatima_aku_edu/Documents/Desktop/Revision%202%20plos%20one%20safe%20trasnfer/Academic%20Editor_Response.pdf)].

We appreciate the Editor’s effort in seeking additional reviewer input to ensure a balanced and fair evaluation. Accordingly, we will proceed to address the remaining reviewer and editor comments to further improve the manuscript.

**Reviewer #2:**

**Comment:**

Review Comments to the Author
Thank you for the opportunity to review this well-conceived and practical quality improvement study, “Rapid response and safety optimization: enhancing critical transfers from outpatient clinics to high acuity areas – a quality improvement project.” The manuscript addresses an important and underexplored aspect of patient safety in low-resource healthcare settings, specifically, the optimization of intra-hospital transfers from outpatient clinics to high-acuity areas.

Overall, this is a technically sound, well-executed project that demonstrates a clear improvement in staff knowledge, structured communication, and aspects of patient transfer safety following the intervention. The manuscript is aligned with PLOS ONE’s scope, and the conclusions are appropriately drawn from the data presented. Below, I provide feedback to help enhance the manuscript further:

Strengths:
1. Timely and Relevant Topic: The focus on critical transfers from outpatient departments, particularly in a low- and middle-income country (LMIC), fills a noticeable gap in current literature and quality improvement discourse. The study is one of the few (and possibly the first) to focus specifically on improving intra-hospital transfers from outpatient departments in a tertiary LMIC setting.
2. Methodological Rigor: The quasi-experimental design, structured intervention (early warning criteria + handoff tool), and longitudinal data collection over a one-year period are appropriate and clearly described.
3. Operational Impact: The intervention is practical, feasible, and shows evidence of integration into routine clinical practice, which is a critical element of QI sustainability.
4. Ethical Considerations: The manuscript notes ERC exemption and informed consent, which is appropriate.

**Recommendations for Improvement:**

**1. Contextualisation and Literature Framing**
- Consider strengthening the Introduction to better highlight the gap in current research specific to outpatient-to-ED transfers. A brief contrast with inter-hospital or inpatient transfers in previous studies would underscore the manuscript’s novel contribution.

**2. Clarity Around Causal Inference**
- The current discussion frames the outcomes as improvements following the intervention, which is valid. However, given the lack of a control group, the language should be cautious in claiming causation. I suggest adding a brief note about this limitation in the Discussion section to strengthen the manuscript’s objectivity. Additionally, the Discussion occasionally restates earlier points without adding new insight.

**3. Implementation Insights and Sustainability**
- The manuscript mentions plans for digitisation and integration of handoff documentation. I recommend briefly describing any early steps toward this (if applicable) or a roadmap for future digitisation efforts to further highlight the potential for scale-up and sustainability.

**4. Presentation and Writing Style**
- The manuscript is generally clear and readable. I believe that minor grammatical corrections are needed, such as the consistency in terms like “handoff” vs. “handover”.
- Consider reducing redundancy in the Discussion and Conclusion sections, where some findings are restated without additional interpretation.
- I recommend referencing and providing descriptions for figures and tables, for instance, “Table 4 shows demographic breakdown…”.

**5. Data Transparency**
- The authors have adequately complied with PLOS ONE’s Data Availability Policy. The manuscript and supporting files contain sufficient data to validate and replicate key findings. Although raw individual-level data are not included, the aggregate and descriptive statistics are appropriately detailed.

**6. Statistical Analysis**
- The use of paired t-tests and descriptive analysis is appropriate. Assumptions were checked (Shapiro-Wilk test), and confidence intervals were reported. However, the interpretation of effect size or clinical relevance for some metrics, like the change in HR/RR, could be discussed further in the Results or Discussion.

Conclusion
This is a valuable contribution to the literature on patient safety and health systems strengthening in LMICs. The authors have implemented a feasible and impactful intervention. It was a good read.

**Reply:**

We sincerely thank the reviewers for their thoughtful, encouraging, and constructive feedback, and for recognizing the value and relevance of our work. We greatly appreciate the time and effort taken to provide detailed recommendations for improvement. We address each point below and have incorporated the suggested changes into the revised manuscript (with tracked changes provided).

1. **Contextualization and Literature Framing**

In the Introduction, we have added **paragraphs 2 and 3** to incorporate relevant literature on inter- and intra-hospital transfers. Despite an extensive search, we were unable to identify any published studies specifically examining transfers from outpatient or clinic settings to emergency departments in low- and middle-income countries (LMICs) or South Asia, including Pakistan. Existing literature from the region largely focuses on inter-facility (hospital-to-hospital) transfers, ambulance and prehospital services, and the epidemiology of emergency department presentations, but does not address outpatient-to-ED transfer processes or outcomes.

Please note that we are referring to paragraph and line numbers rather than absolute line numbers, as the latter may differ between the clean and tracked-changes versions.

2. **Clarity Around Causal Inference**

We are making a clear statement in the **limitation section** of the discussion **(4^th^ line)**. We have revised the Discussion to minimize repetition and ensure that each point provides new insight or interpretation of the findings.

3. **Implementation Insights and Sustainability**

We have added a brief description of early steps and plans for digitization and integration of handoff documentation in the Discussion **(7th paragraph, line 8)** to highlight the potential for scale-up and sustainability.

4. **Presentation and Writing Style**

We have made every effort to reduce grammatical errors. As mentioned above, we have revised the entire Discussion/Conclusion and added additional interpretations. Furthermore, we have referenced and provided descriptions for all figures and tables while ensuring compliance with PLOS ONE requirements.

5. **Data Transparency**

We have provided complete data, which is included in the file inventory, including all pre- and post-test data as well as the patient dataset. Should you require any additional specific information, we would be happy to provide it.

6. **Statistical Analysis**

We appreciate the reviewer’s comment. The significance of effect sizes has been added to the **Results section under the pre-test and post-test headings** **(Paragraph 2)**.

Additionally, the interpretation of changes in HR and RR has been added under **'Outcomes of patient transfers following the implementation of the quality improvement initiative**' in the results section **(Paragraph 2)**.

**Reviewer #3**

**Comment:**

Minor calculation mistake in line number 301, the percentage of female nursing staff should be 77.5 instead of 80. and in line number 318: table 4. row number 3 and 4, 2nd column Female % should be 77.5 and Male 22.5.

Therefore, need this to be corrected. Rest seems OK.

**Reply:**

Thank you very much for your careful review and kind guidance. We sincerely appreciate you pointing out the calculation errors. I have now corrected the percentages as recommended.

**Reviewer #4**

**Comment:**

This manuscript was written in an intelligible manner, using the acceptable standard of English. However, minor corrections such as punctuation marks and grammar should be modified thoroughly.

Regarding publication ethics, even though this is a QI study, PLOS ONE requires ethics info. You can write:
“This quality improvement study was approved as a non-research activity by the Ethical Review Committee of Aga Khan University Hospital, which waived the need for informed consent as no patient-identifiable data was used.”
It will also be important to mention the study code and the date on which the ethical clearance was issued.
In case you did not receive the ethical clearance, you must explain why you did not obtain formal ethics approval and under what guidelines it was exempted.

No proof of dual publication was noted.

**Reply:**

We thank the reviewer for the suggestion and have corrected grammatical and punctuation errors throughout the manuscript.

Regarding publication ethics, we would like to clarify that, in accordance with institutional policy at Aga Khan University Hospital, all research and quality improvement projects undergo Ethical Review Committee (ERC) oversight. For this project, permission was first obtained from the Chief Medical Officer as well, and the ERC subsequently granted an exemption. No patient-identifiable data was used, and written informed consent was obtained from participating nurses and healthcare staff for pre- and post-test assessments. The ERC exemption letter is included in the inventory files.

While we appreciate the reviewer’s suggested wording, we have retained the phrasing above to accurately reflect our institutional process and policies. We have also added a statement confirming that this manuscript is original, has not been published elsewhere, and no dual publication has occurred.

**Reviewer #4: Reviewer 4 additional comments from the Word document sent as an attachment:**

**General Comments**

This is a well-organized and useful Quality Improvement (QI) project that aimed to improve the safety and effectiveness of patient flow from outpatient clinics to the ED at a tertiary care setting in Pakistan. The subject is especially pertinent in low- and middle-income countries (LMICs), where healthcare systems operate under varying challenges specific to each setting. The authors discuss a topic that is often overlooked when considering patient safety, and the interventions described are relatively pragmatic and potentially scalable. However, there are several areas where improvements would enhance the manuscript's scientific rigor, clarity, and impact.

**Specific Comments**

1. **Title & Abstract**

- The title is well structured, but could be concise, such as

*“Improving the Safety of Outpatient to Emergency Department Transfers: A Quality Improvement Study in a Tertiary Hospital in Pakistan”*

- The abstract follows a logical structure, yet needs keywords like **quality improvement,** **handover,** and **patient safety** to improve search results

2. **Introduction**

- The similar initiatives as part of the literature supporting the relevance of your study were not cited sufficiently, such as QI initiatives in similar LMIC settings (abroad and local).
- Please look for studies in QI Initiatives conducted in similar settings to support your argument.

- Reword or rephrase for conciseness and impact, maybe to write:

*‘Transfers from outpatient clinics to the Emergency Department are vulnerable points in the care continuum, especially in low and middle-income countries, where the standardized protocols are often lacking’*

- In this introduction, the problem and its significance were not clearly defined.
- Please define clearly the problem and its significance.
- Secondly, support your problem by mentioning the available data on occurrences, like noting that:

*‘? % of patients reported missing paperwork…’*

- It is always recommended that your introduction ends with the purpose or aim of conducting the study, e.g.:
  *"This study aimed to improve the safety, documentation, and tracking of patients transferred from outpatient clinics to the ED through a quality improvement approach at Aga Khan University Hospital.*

3. **Methods**

**3.1 Study Design and Setting**

This is not a clinical trial. Therefore, mention that this is a QI initiative to minimize confusion for the readers.

When conducting a Quality Improvement study in healthcare, the SQUIRE 2.0 (Standards for Quality Improvement Reporting Excellence) is often used for reporting.

SQUIRE 2.0 provides a framework for authors to report their QI projects clearly and transparently.

Therefore, I recommend that you use the SQUIRE 2.0 structure to address the following 3 areas, namely;

a) ***Context***

Describe the hospital setting (for instance, yearly patient numbers, number of specialties)

b) ***Intervention rationale:***

Explain why these specific tools, such as the **transfer form** and **clinic attendant**, were chosen.

c) ***Measures:***

Lastly, using the SQUIRE 2.0 structure, define: ***process*** vs ***outcome*** vs ***balancing measures***.

**3.2 Intervention Design**

Though you expressed in detail the interventions applied in your study, it is unclear how you designed the interventions. Explain how you designed the interventions, such as Process mapping, Stakeholder meetings, and the PDSA cycle (PLAN-DO-STUDY-ACT)

**3.3 Data Collection and Measures**

- This research (QI Intervention study), referencing primary, secondary, and process measures, is required to understand the intervention's effectiveness.

In this case, primary measures (number of adverse events or patient harm during transfers), secondary measures (triage accuracy, length of ED stay, or patient satisfaction), and process measures (form usage, adherence to protocol, or documentation completeness)

Please address this shortcoming in your data collection section.

- The explanation of how the audits were conducted was also not provided.

It will be important to mention this in the data collection section.

4. **Ethics Statement**

Even though this is a QI study, PLOS ONE requires ethics info. You can write:

“*This quality improvement study was approved as a non-research activity by the Ethical Review Committee of Aga Khan University Hospital, which waived the need for informed consent as no patient-identifiable data was used.”*

It will also be important to mention the study code and the date on which the ethical clearance was issued.

In case you did not receive the ethical clearance, you must explain why you did not obtain formal ethics approval and under what guidelines it was exempted.

5. **Results**

Though the data was presented in table form extensively, to strengthen the QI focus, I recommend that you also include ***run charts*** or ***control charts***.

6. **Discussion**

In your first paragraph, summarize the main results of the study, followed by how your intervention brought about change.

- Though the discussion section articulated deeply on challenges, critical challenges such as staff resistance, staff turnover, buy-in, and form fatigue, among others, did not come out.

I therefore recommend that you add one paragraph to discuss these challenges explicitly.

- Since this is a QI intervention study, this study lacks comparison with other similar local or international published QI Intervention studies.

Therefore, I recommend that you compare your results with other published QI interventions in similar settings, such as the WHO Safer Handover toolkits or other LMIC QI initiatives. This comment is also mentioned in your introduction!

- It would be prudent to suggest explicitly the areas for further improvement, such as digital tools, automated alerts, and sustainability audits. This would ensure continuity and improve future interventions.

Outline clearly the practical and actionable future activities that would enhance the safety of the outpatient during emergency transfers.

7. **Conclusion**

The current version is good but consider making sustainability and scalability more explicit. Keep it concise and focused on impact by including the following phrase:

*“Structured communication, physical accompaniment, and stakeholder collaboration significantly improved the safety of patient transfers from outpatient clinics to the ED. This approach is low-cost, sustainable, and scalable to similar settings.”*

**Reply:**

We thank the reviewer for these constructive comments.

Please note that we will be referring to paragraph and line numbers rather than absolute line numbers, as the latter may differ between the clean and tracked-changes versions in the below reply.

1. **Title & Abstract**

We agree with the suggestion for conciseness and have revised the title accordingly:
“**Improving the Safety of Outpatient to Emergency Department Transfers: A Quality Improvement Study in a Tertiary Hospital in Pakistan**.”

We have added the following keywords to enhance visibility and searchability*:* Ambulatory care, handoff, patient safety, quality improvement.

2. **Introduction:**

Regarding the comment that similar initiatives as part of the literature supporting the relevance of our study were not cited sufficiently, such as QI initiatives in similar LMIC settings (abroad and local), we conducted a thorough search but were unable to find any local studies or studies from LMICs specifically addressing outpatient-to-emergency or high-acuity transfers. However, we have cited similar studies that have addressed related aspects of patient transfers. In addition, we have included literature on both intra-hospital and inter-hospital transfers from LMICs as well as other settings to better support the relevance of our study.

We thank the reviewer for providing an impactful suggestion; we have retained the statement as recommended and have rephrased the introduction for conciseness and clarity, clearly defining the problem and its significance.

While we would have liked to include data on occurrences, such as missing paperwork, there was no established process to track or streamline these events; this limitation has been noted under the Context heading **(2nd paragraph, line 9).**

Finally, we have explicitly stated the study aims under the heading “**Objectives”**.

3. **Methods**

**3.1 Study Design and Setting**

The Methods section has been revised to emphasize that this was a Quality Improvement (QI) initiative and not a clinical trial, in order to avoid reader confusion.

According to your recommendation, we have aligned the manuscript with the SQUIRE 2.0 framework along with the following.

a) **Context:**

We have provided details of the hospital setting, including patient volumes and range of specialties, under the Methodology section **(Context).**

b) **Intervention rationale:**

We have addressed this in the manuscript, providing the rationale for choosing specific tools, including the transfer form and clinic attendant, in the **Context** **(paragraph 2)** and **Discussion sections** **(paragraph 3, line 18).**

c) **Measures:**

A dedicated Measures section has been added to the manuscript **before the ethical consideration section**. This section now explicitly describes the process, outcome, and balancing measures.

**3.2 Intervention Design**

In the manuscript, we have described the “Plan” phase of the PDSA cycle in the **Quality Improvement Initiative**, and we have also tried to outline the “Do,” “Study,” and “Act” phases across the different phases of the quality improvement initiative.

3.3 **Data Collection and Measures**

We have included the primary, secondary, and process measures in the manuscript under the heading “**Measures,**” and have also provided a brief description of how audits were conducted.

4. **Ethics Statement**

This has already been addressed in the response provided above.

5. **Results**

We have added a control chart in the **Results section**, which demonstrates the transfer patterns and their significance with Upper and Lower Control Limits (UCL and LCL).

6. **Discussion**

We appreciate the reviewer’s detailed suggestions. In the revised manuscript, we have:

- We have added a summary in the 1^st^ paragraph of the discussion section, with how our quality initiative brought change.
- Added a paragraph in the Discussion explicitly addressing the challenges encountered during the initiative **(paragraph 8).**
- We have added literature on inter- and intra-hospital transfers. While we did not find studies from LMICs or South Asia specifically, we have cited other studies on transfers from outpatient to ED settings. Additionally, we have included a discussion of the WHO Safer Handover toolkits **(paragraph 3, line 22)** to provide context and comparison for our QI intervention.
- Suggested actionable areas for future improvement, including the implementation of digital tools and periodic sustainability audits, to enhance the continuity and safety of outpatient-to-ED transfers **(paragraph 7, line 7).**

7. **Conclusion**

We have revised the conclusion and added the advised statement to the conclusion.

**Reviewer #5**

Good day
Thank you for asking me to review the clinical paper
Overall, the manuscript presented a very important study aiming at improving the care and safety of patients in acute care episode. However, the manuscript has not been well arranged and the chosen study design was not properly followed
1. It was not clear whether the design is quazi experimental study, descriptive cross sectional or retrospective medical record review. The manuscript has characteristics depicting all the three
2. It is not clear whether this is a pilot study or a full scale study conducted after the preliminary pilot study
3. Pre and post quazi experimental design was mixed up, the intervention period is not clear. Especially, phase II consist of both intervention and outcome evaluation
4. many tools were mentioned and the effectiveness of the tools for early detection of patient deterioration were not discussed
5. Post intervention evaluation was not clear as there was no comparison between the pre and post. Therefore, results were ambiqous
6. The tables are many and mechanical. It does not show evidence of quazi experimental study conducted. It was mere listing of materials/instruments
7. Study setting, population, sample size and procedure for data collection not clear
8. How confounding variables were controlled not discussed
9. There was no clear evidence of testing a particular tool effectiveness.
10. Vital signs are known parameters to determine the degree of wellness or illness of patients. It is not appropriate to test vital sign usefulness or effectiveness. We know already

Summary
I. Re visit the study design
2. Discuss the baseline assessment only in phase I and the assessment tool to test their/its efficacy
3. Discuss the Interventions
4. Discuss the outcome evaluation to determine the effectiveness of the applied tools
Compare your results pre and post and make recommendations
5. Most of the tables presented are not necessary. Present the pre assessment (Phase 1)tables, the Interventions tables, post intervention table. Also present tables on association or correlation between the post and interventions etc
6. Describe each of the research step clearly: The population, sampling, sample size, eligibility criteria, validity, reliability etc
7. You may combine few related results/tables
8. Make your discussion coincide, identify what this study add or concluded, compare with previous studies, state the strength and limitations of the study

**Reply to** **Reviewer’s general comments:**

Thank you for your detailed review and for highlighting areas that required clarification. We acknowledge that the initial presentation may have caused confusion regarding the study design and structure. In response, we have made the following key revisions:

1) Thank you very much for pointing this out. We agree that it may have caused confusion, so we have clarified the study design. We are also stating it here:

*This quality improvement project was carried out in two phases. The pre-implementation phase involved development and pilot testing of the tools, while the implementation phase focused on hospital-wide rollout through structured staff training. Training effectiveness was evaluated using a quasi-experimental, one-group pretest–posttest design in a subset of staff selected through convenience sampling.*

*In parallel, after the initiative was fully implemented, a prospective observational study was undertaken to evaluate the transfer process in practice and assess associated outcomes. The study population included critically ill adult patients (≥18 years) requiring transfer from outpatient clinics to high-acuity areas, as identified by the treating physician and formally referred to the emergency department. Outpatient nursing staff responsible for facilitating transfers were also included.*

2) We have revised the manuscript to clearly distinguish the pre-implementation phase, which included tool development and pilot testing, from the post-implementation phase, which involved nurse training, pre-test/post-test evaluation, hospital-wide rollout, and outcome evaluation.
3) In response to the reviewer’s observation, we have added separate headings and included a flow diagram to clearly represent both phases, explicitly delineating the quality initiative period/intervention and the outcome evaluation in Phase II. See Figure 3.
4) In response to the reviewer’s comment on the effectiveness of the tools, we have added a dedicated ‘Measures’ section describing the outcome indicators used to assess early detection of patient deterioration. As it was an observational study and did not have a control group, it does not allow for causal inference. The consistently favorable outcomes across multiple domains over a 12-month period suggest that the revised early signs and SBAR handoff contributed to safer patient transfers and more reliable communication. We have now revised the Discussion section to explicitly highlight this interpretation and have acknowledged the lack of a control group and reliance on retrospective data as study limitations.
5) We acknowledge the reviewer’s comment. As this study did not include a pre-intervention/pre-quality initiative comparison group, a formal pre- and post-analysis was not possible. Outcomes were prospectively observed during the quality improvement period and are presented accordingly. It is important to note that, before this initiative, there was no established process for transfers from outpatient to emergency care, so there was no available data to compare it with. This is the limitation of the study and has been clearly mentioned in the discussion section under the heading **Limitations.**
6) We agree with the reviewer’s comment. The tables in our manuscript were primarily designed to summarize the clinical characteristics and outcomes. A control group was not included, as it was difficult to add one due to ethical considerations. We have clarified this point in the revised manuscript and highlighted it in the Limitations section.
7) We have revised the Methodology section to provide greater clarity on the study setting, population, sample size, and data collection procedures (see section Study Design and Population).
8) We appreciate the reviewer's comment regarding confounding variables. However, as our study aimed to provide a descriptive overview of the data, we did not perform any inferential statistical analyses or modeling that would require controlling for confounding variables. Our primary focus was on summarizing the characteristics of the study population and describing the distribution of variables over time. Therefore, we did not consider it necessary to adjust for confounding variables in this descriptive analysis.
9) We acknowledge the reviewer’s concern regarding the testing of tool effectiveness. The content validity of the tools was established through the modified Delphi method. In addition, the quality improvement initiative was piloted in the Medicine outpatient clinic to assess feasibility, reliability in practice, and the practical application of the revised early warning criteria, the standardized transfer process flow, and the SBAR handoff tool. Staff feedback on clarity, usability, and workflow integration was incorporated to refine the tools for consistent use and smooth hospital-wide adoption. These steps are now described more clearly in the manuscript under the pre-implementation phase. We note that reliability testing using psychometric analysis was not conducted; while this was initially mentioned, we have now clarified these steps and their scope in the revised manuscript.
10) We agree that vital signs are well-established indicators of patient wellness and illness, and therefore, their usefulness does not require further testing. In our initiative, instability was identified in the outpatient clinic, and in 24.3% of cases, staff-initiated interventions based on patients’ vital signs before transfer. Comparison of pre- and post-intervention vital signs demonstrated stabilization and clinical improvement in these patients, indicating the effectiveness of timely recognition and management. We believe this observation adds value to the manuscript, but if the reviewer feels it should be removed, we are willing to do so.

**Reply to the Reviewer’s summary section:**

1. The study design has been restated for clarity.
2. Tool development and piloting are now described under the pre-implementation phase, while subsequent steps are detailed in the implementation phase.
3. Details of the intervention has been under the heading “**Quality improvement initiative”** and a separate **“Measures”** section has been added. The phases of the study are now presented with greater clarity.
4. As noted, this study did not include a control group or retrospective data for comparison, and this limitation has been explicitly acknowledged in the manuscript. We respectfully ask that the practical challenges of implementing this quality improvement work be considered when evaluating this aspect.
5. We have merged some of the tables for clarity and removed Table 1. As this was an observational study, we did not have retrospective data to formally compare patient outcomes before and after the quality improvement period. However, a pre- and post-test was conducted to assess the effectiveness of staff training. The result tables now present the pre- and post-test for training effectiveness, demographic characteristics of the study population, changes in vital signs before and after transfer, and outcomes, including mortality, length of stay, disposition, etc.
6. Each step of the process has been described in detail, and a diagram has been added to illustrate the pre-implementation and post-implementation phases.
7. Related tables have been combined for clarity.
8. The Discussion section has been revised to align more closely with the study objectives and results. Specifically:
   - The contribution of this initiative to literature is highlighted, especially its novelty in adapting early warning signs, structured transfer processes, and standardized handoff communication tools for outpatient settings in our context.
   - Comparisons with previous studies on patient transfer safety and early recognition of deterioration are provided.
   - Strengths such as multidisciplinary tool development, piloting for feasibility, and hospital-wide implementation are emphasized.
   - Limitations—including absence of pre-implementation data, reliance on post-implementation measures, and lack of formal psychometric reliability testing—are clearly mentioned.
