# Supplementary material for: Improving the safety of outpatient to Emergency Department transfers: A quality improvement study in a tertiary hospital in Pakistan
Source: PLoS One. 2025 Nov 5;20(11):e0336347. doi: 10.1371/journal.pone.0336347 (PMC12588536; doi:10.1371/journal.pone.0336347)
Supplement: Attachment — Submitted filename: Reviewers Comments_2nd review_22.10.2025.docx [file pone.0336347.s007.docx]

**General Comments**

This is a well-organized and useful Quality Improvement (QI) project that aimed to improve the safety and effectiveness of patient flow from outpatient clinics to the ED at a tertiary care setting in Pakistan. The subject is especially pertinent in low- and middle-income countries (LMICs), where healthcare systems operate under varying challenges specific to each setting. I have no further objections to the quality of this study. I therefore recommend PLOS ONE to publish this impactful work.

**Specific Comments**

1. **Title & Abstract**

*The reviewer is satisfied with the quality of the responses to this section, particularly the modification of the title.*

2. **Introduction**

- The explanation from the authors about the unavailability of similar studies conducted in other LMIC settings is understandable and acceptable.
- The problem and significance were clearly outlined in the introduction.

3. **Methods**

**3.1 Study Design and Setting**

The distinction about between the Clinical Trial and QI initiative has improved the conciseness of this study.

The introduction of SQUIRE 2.0 improved transparency (Context, Intervention rationale and Measure were sufficiently addressed)

4. **Ethics Statement**

The study code, date of issue of the ethical clearance, and the issuing body were all clearly explained.

5. **Results, discussion , and conclusion**

The recommendations were correctly addressed and elaborated on the depth of the study’s impact in the LMIC
